# Hidden cost of disease in a free‐ranging ungulate: brucellosis reduces mid‐winter pregnancy in elk

**DOI:** 10.1002/ece3.4521

**Published:** 2018-10-28

**Authors:** Gavin G. Cotterill, Paul C. Cross, Arthur D. Middleton, Jared D. Rogerson, Brandon M. Scurlock, Johan T. du Toit

**Affiliations:** ^1^ Department of Wildland Resources Utah State University Logan Utah; ^2^ U.S. Geological Survey Northern Rocky Mountain Science Center Bozeman Montana; ^3^ Department of Environmental Science, Policy and Management University of California Berkeley California; ^4^ Wyoming Game and Fish Department Pinedale Wyoming

**Keywords:** *Brucella abortus*, *Cervus canadensis*, disease ecology, Greater Yellowstone Ecosystem, pregnancy‐specific protein‐B, winter feedgrounds

## Abstract

Demonstrating disease impacts on the vital rates of free‐ranging mammalian hosts typically requires intensive, long‐term study. Evidence for chronic pathogens affecting reproduction but not survival is rare, but has the potential for wide‐ranging effects. Accurately quantifying disease‐associated reductions in fecundity is important for advancing theory, generating accurate predictive models, and achieving effective management. We investigated the impacts of brucellosis (*Brucella abortus*) on elk (*Cervus canadensis*) productivity using serological data from over 6,000 captures since 1990 in the Greater Yellowstone Ecosystem, USA. Over 1,000 of these records included known age and pregnancy status. Using Bayesian multilevel models, we estimated the age‐specific pregnancy probabilities of exposed and naïve elk. We then used repeat‐capture data to investigate the full effects of the disease on life history. Brucellosis exposure reduced pregnancy rates of elk captured in mid‐ and late‐winter. In an average year, we found 60% of exposed 2‐year‐old elk were pregnant compared to 91% of their naïve counterparts (a 31 percentage point reduction, 89% HPDI = 20%–42%), whereas exposed 3‐ to 9‐year‐olds were 7 percentage points less likely to be pregnant than naïve elk of their same age (89% HPDI = 2%–11%). We found these reduced rates of pregnancy to be independent from disease‐induced abortions, which afflict a portion of exposed elk. We estimate that the combination of reduced pregnancy by mid‐winter and the abortions following mid‐winter reduces the reproductive output of exposed female elk by 24%, which affects population dynamics to a similar extent as severe winters or droughts. Exposing hidden reproductive costs of disease is essential to avoid conflating them with the effects of climate and predation. Such reproductive costs cause complex population dynamics, and the magnitude of the effect we found should drive a strong selection gradient if there is heritable resistance.

## 1 INTRODUCTION

Acute infections that increase host mortality garner broad interest. By virtue of their pathogenicity, they can locally threaten species persistence (Frick et al., 2015; McCallum, 2012) and trigger disturbances that reverberate through the ecosystem (Holdo et al., 2009; Hollings, Jones, Mooney, & Mccallum, 2013). By comparison, the effects of chronic infections are understudied. Parasites of low pathogenicity certainly have the potential for population‐level effects (McCallum, 1994), but isolating the effects of chronic disease from other environmental stressors requires intensive long‐term study (Cross et al., 2009; Gorsich, Ezenwa, Cross, Bengis, & Jolles, 2015; Jolles, Cooper, & Levin, 2005). Chronic diseases may impact host reproduction either directly by reducing pregnancy or causing abortions, or indirectly by reducing energy reserves and body condition. In both cases, the importance of disease may be underappreciated. As a case‐in‐point, some elk (*Cervus canadensis*) herds in the Greater Yellowstone Ecosystem (GYE) of the western United States have a high prevalence of brucellosis—a chronic reproductive disease—yet the costs to elk have long been considered “unimportant as a practical matter” (National Research Council, 1998). This is despite the hallmark symptom of the pathogen, and its primary means of transmission, being abortion (Thorne, Morton, Blunt, & Dawson, 1978). In the GYE, brucellosis is caused by the bacterium *Brucella abortus* and is routinely detected in elk, bison (*Bison bison*), and occasionally domestic cattle. Significant reductions in bison reproduction and recruitment have been attributed to this disease (Fuller et al., 2007; Geremia et al., 2008; Hobbs et al., 2015), but attempts to formally characterize the reproductive consequences of this disease in elk have lagged. There is no evidence that brucellosis affects survival in bison (Fuller et al., 2007) or elk (Benavides et al., 2017), and limited captive trials with elk in the 1970s suggested that major reproductive costs were limited to the firstyear post‐infection (Thorne et al., 1978). More recently, Oldemeyer, Robbins, and Smith (1990) observed nonsignificant declines in calf production by seropositive female elk, while Foley, Cross, Christianson, Scurlock, and Creel (2015) were unable to detect changes in calf:cow ratios in herds where prevalence for the disease was comparatively high.

Elk vital rates in the GYE have been the subject of intense scrutiny since the reintroduction of gray wolves (*Canis lupus*) in the 1990s, which has also coincided with increasing brown bear (*Ursus arctos*) (Schwartz, Haroldson, Gunther, & Moody, 2006) and cougar (*Puma concolor*) numbers (Clark, Rutherford, & Casey, 2013). As with many ungulate species, adult elk survival is generally high so that population dynamics are largely driven by variable recruitment (Cole et al., 2015; Middleton, Kauffman, McWhirter, Cook et al., 2013; Raithel, Kauffman, & Pletscher, 2007) and numerous studies have evaluated the relative influences of top–down and bottom–up mechanisms on elk productivity and abundance (Creel, Christianson, Liley, & Winnie, 2007; Mech, Smith, Murphy, & MacNulty, 2001; Middleton, Kauffman, McWhirter, Jimenez et al., 2013; Proffitt, Cunningham, Hamlin, & Garrott, 2014; White et al., 2011). Reduced elk pregnancy due to nutritional limitation is well established (Cook et al., 2004) as a demographic response to adverse climatic conditions. For example, a 7% difference in yearling pregnancy and 15% difference in adult pregnancy have been attributed to winter severity and reduced summer precipitation, respectively (Proffitt et al., 2014). Similarly, a 4‐year, 19% decline in pregnancy among migrant elk was found to be largely driven by poor reproduction of young and lactating females (Middleton, Kauffman, McWhirter, Cook et al., 2013). More controversially, studies have attributed herd‐level variation in elk pregnancy to the indirect costs of predation acting through increased vigilance and predator avoidance. One study attributed as much as a 43% relative decline in elk pregnancy to the stress‐induced nonconsumptive effect of wolf presence (Christianson & Creel, 2014), while others found no evidence for such an effect (Proffitt et al., 2014; White et al., 2011). Brucellosis may have been at low levels or even absent when and where many of these studies were conducted and, as such, the effects of this disease were not investigated. In recent years, however, seroprevalence for brucellosis among elk has been increasing and expanding to new areas around the GYE (National Academies of Sciences, Engineering, and Medicine, 2017) so that ignoring its effects may no longer be tenable. Furthermore, calibration for the full effect of brucellosis will be essential if the well‐studied GYE elk population is to be used as a model for understanding the effects of climate and predation on other elk populations or ungulate species.

**Figure 1:**
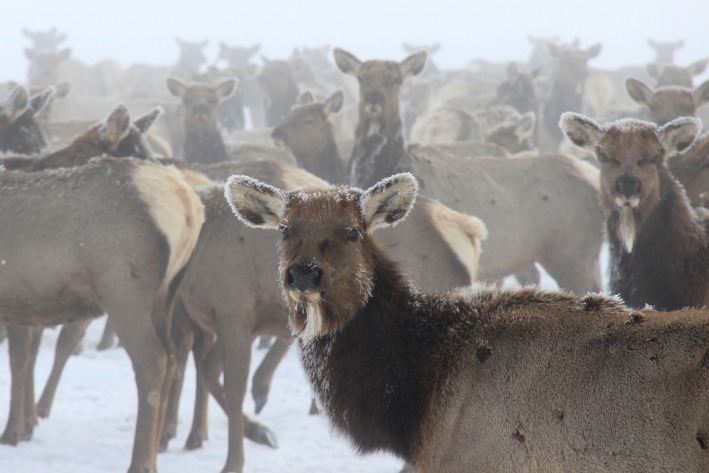
Elk at a winter feedground in Wyoming. Photo credit: Mark Gocke, WY Game & Fish Dept

Historically, supplemental winter feedgrounds for elk in the GYE have been focal points for brucellosis contagion (Figure 1). In the Wyoming portion of the southern GYE, there are 23 feedgrounds operating annually and, at times, local seroprevalence can reach as high as 60% (Cotterill et al., 2018). With such high exposure, one might expect that abortions should be frequent and readily observed, but on average, fewer than two fetuses were detected annually over a 50‐year period (Cross et al., 2015). Because of the difficulty of observation, a more rigorous approach for detecting abortions was undertaken using vaginal‐implant transmitters (VITs) which were cultured for *B. abortus* within days after being expelled due to abortion or parturition. A comprehensive study found that, on average, 16% of seropositive and pregnant elk abort per year (Cross et al., 2015) which is consistent with previous findings (Thorne et al., 1978). Use of VITs also confirmed that most abortions occur late in pregnancy (March‐May), which is consistent with *Brucella* spp. across host species (Jamil et al., 2017). The shortcoming of this methodology lies in its potential to underestimate the total reproductive cost of the disease. VITs are designed to be expelled by the passage of a fetus through the birth canal and so are only inserted into those animals determined to be pregnant through ultrasound. Because VITs are inserted in winter (January and February), this method excludes any elk that either failed to conceive in the previous autumn or else lost the pregnancy early in gestation.

Here, we report on a study of mid‐ and late‐winter age‐specific pregnancy rates and brucellosis serology for elk in free‐ranging herds that attended supplementary feedgrounds in the GYE spanning two decades. Contrary to the conventional assumption that the only effect of brucellosis on elk reproduction is spontaneous induction of late‐term abortion (National Research Council, 1998), we found exposed females to also incur a substantial risk of lost reproductive opportunity prior to when abortions occur. As an individual must first be pregnant in order to have an abortion, we assumed that the difference in pregnancy and abortion losses are additive when calculating the total reproductive costs of disease. Taken together, these double what was previously thought to be the total reproductive cost of brucellosis to elk.

## 2 MATERIALS AND METHODS

### 2.1 Study area and data collection

Data were collected in western Wyoming, south of Yellowstone National Park, USA, where supplementary winter feedgrounds are used by approximately 80% of the region's elk (Dean et al., 2004). The National Elk Refuge in Jackson, WY, is operated by the US Fish and Wildlife Service (USFWS) and 22 additional feedgrounds are operated by the Wyoming Game and Fish Department (WGFD; see Cotterill et al., 2018). Our data were collected by the WGFD for research and management purposes across all 23 feedgrounds and 2 nearby unfed wintering locations between 1995 and 2017. All captured elk receive permanent ear tags, which enable identification at subsequent recaptures, and this allowed us to track a subset of individual elk over time. Age, pregnancy, and brucellosis serostatus were known for 1,236 records of female elk. All serology and pregnancy data were collected between January 3 and April 15, with 90% of data collection occurring prior to March 1, of any year.

### 2.2 Covariates

Age ranged from 1½ to 19½ years at the time of capture and was determined either through recapture of animals marked as calves or yearlings (when elk are morphologically distinct, *n* = 824) or through cementum annuli analysis (Matson's Lab, Milltown, MT, USA) of a vestigial canine taken from new captures (*n* = 412). Yearlings were excluded from most pregnancy testing for logistical reasons; testing is expensive, and elk generally do not reach reproductive maturity until age two, meaning that only one in about five female yearlings might be expected to be pregnant. As such, the yearling age class is poorly represented (*n* = 18). Two‐year‐olds were the best‐sampled age and accounted for nearly 25% of the data. Sample size decreased with age and animals of 10 years or older accounted for only 10% of the data.

The collection of serological data from GYE elk has been described elsewhere (Cross, Edwards, Scurlock, Maichak, & Rogerson, 2007; Maichak et al., 2017; Scurlock & Edwards, 2010). Briefly, to obtain blood samples and teeth for cementum analysis, baited corral traps and/or chemical immobilization were used. Serological assays were conducted and interpreted using current National Veterinary Services Laboratories protocols. Serological profiles were categorized using the United States Department of Agriculture's brucellosis eradication uniform methods and rules for cervids (APHIS 91‐45‐013), resulting in a binary (seronegative or seropositive) determination. In a secondary analysis, fluorescent polarization (FP) assay results (in millipolarization units) were included as a continuous variable (Gall et al., 2001).

Pregnancy status was determined either by transrectal ultrasound at the time of capture (*n* = 260) or a blood test for pregnancy‐specific protein‐B (PSPB, *n* = 976) performed by BioTracking, Inc., Moscow, ID, USA, and previously validated in elk with 97% accuracy (Noyes, Sasser, Johnson, Bryant, & Alexander, 1997). If both ultrasound and PSPB results were available for the same animal and capture, we took PSPB as the more accurate indicator.

Wyoming Game and Fish Department performs ground counts at each of their feedgrounds in peak winter. From these, we were able to construct calf:cow ratios for each feedground. In the case of four locations, we also had adequate time‐series data for seroprevalence with which to test for a relationship between disease prevalence or changes in prevalence and calf:cow ratios. Due to the timing of transmission (abortion) events and the length of time required to develop a titer (test seropositive following exposure), it is not perfectly clear whether the change in seroprevalence at a site from the previous year (*t−1* to *t*) or the change from 2 years previous (*t−2* to *t−1*) should be a stronger predictor of current year (*t*) calf counts. Therefore, we tested both, along with seroprevalence in the previous year (*t−1*) and 2 years prior (*t−2*).

### 2.3 Statistical modeling and evaluation

Using a Bayesian hierarchical approach, we evaluated the effect of serostatus on pregnancy while accounting for variation between age groups, year effects, and possible effects of pregnancy test method. A preliminary analysis determined that binning ages 3–9, as well as 10 or older, while keeping yearlings and 2‐year‐olds separate, yielded the best balance of model fit and simplicity based on information criteria. Location was initially included as a model term but dropped due to insignificant variance in site effect.

Our response variable was the pregnancy status *Y* for individual elk *i* at age *j* in year *k*. We assumed that *Y*_*ijk*_ was a Bernoulli trial with a probability of being pregnant, *p*_*ijk*._ We then used a logit link function to relate the probability of pregnancy to covariates. Let *α*_0_ represent an overall intercept term, where *α*_*j*_ and *α*_*k*_ represent age‐ and year‐specific offsets, respectively. Let *β*_0_ represent the regression coefficient associated with a dummy variable for serostatus, *s*_*i*_, where additional age‐varying effects of serostatus are represented as *β*_*j*_. In a post hoc analysis, we also tested for year‐varying effects of serostatus, represented as *β*_*k*._ Let *ɣ* represent the regression coefficient associated with the dummy variable for pregnancy test method, *m*_*i*_. Thus, the model including all terms takes the form:

$$\text{logit}\left(p_{\mathrm{ijk}}\right)=\alpha_{0}+\alpha_{\mathrm{jk}}+\left(\beta_{0}+\beta_{\mathrm{jk}}\right)s_{i}+\gamma m_{i}$$

Six alternative models were compared against this one to determine the effect of serostatus on pregnancy probability, while accounting for the relative importance of age‐ and year‐specific variation, as well as the effect of pregnancy test method (Table 1).

**Table 1:** Seven models to estimate the effect of serostatus on the probability of pregnancy

Model	Formula	Intercepts	Slopes
1	logit(*p*_*i*_) = *α*_0_ + *β*_0_ *s*_*i*_ + *γm*_*i*_	Fixed	Serostatus; method
2	logit(*p*_*ik*_) = *α*_0_ + *α*_*k*_ + *β*_0_ *s*_*i*_ + *γm*_*i*_	Year‐varying	Serostatus; method
3	logit(*p*_*ij*_) = *α*_0_ + *α*_*j*_ + *β*_0_ *s*_*i*_ + *γm*_*i*_	Age‐varying	Serostatus; method
4	logit(*p*_*ij*_) = *α*_0_ + *α*_*j*_ + (*β*_0_ + *β*_*j*_)*s*_*i*_ + *γm*_*i*_	Age‐varying	Age‐varying serostatus; method
5	logit(*p*_*ijk*_) = *α*_0_ + *α*_*jk*_ + *β*_0_ *s*_*i*_ + *γm*_*i*_	Age‐ and year‐varying	Serostatus; method
6	logit(*p*_*ijk*_) = *α*_0_ + *α*_*jk*_ + (*β*_0_ + *β*_*j*_)*s*_*i*_ + *γm*_*i*_	Age‐ and year‐varying	Age‐varying serostatus; method
7^a^	logit(*p*_*ijk*_) = *α*_0_ + *α*_*jk*_ + (*β*_0_ + *β*_*jk*_)*s*_*i*_ + *γm*_*i*_	Age‐ and year‐varying	Age‐ and year‐varying serostatus; method

a Model 7 was added post hoc to test for a significant disease by year interaction.

Nearly all of our data were collected before March, which we assumed would be prior to when elk typically abort due to brucellosis (Cross et al., 2015). We tested that assumption using seropositive records by modeling pregnancy status as a function of day of the calendar year, collapsing all years. If our results were influenced by early abortions occurring prior to March, then the probability of pregnancy should have decreased over our sampling period. Otherwise, animals in our sample that were not pregnant either failed to conceive during the preceding rut, or suffered intrauterine mortality prior to January. A logistic regression was performed using day of calendar year to predict the probability of pregnancy for seropositive elk with and without age effects.

Within 1,236 records, there were 869 unique individuals. Individual was not included as a variable in our pregnancy models because relatively few animals were recaptured more than twice. However, these longitudinal data provided an additional opportunity to test the hypothesis that infected animals recover from the fertility consequences of brucellosis and that any reductions in pregnancy probability attributed to serostatus would disappear over time. For the subset of recaptured individuals that ever tested seropositive, we created a coarse metric of “time‐since‐infection” by tracking the year in which an individual was first observed to be seropositive (see Supporting information Appendix S2). We then tested whether time‐since‐infection or fluorescent polarization (FP) assay values (when available) were significant predictors of pregnancy status. We also regressed time‐since‐infection against FP values to test whether our data support the belief that FP values decline in individuals as elk lose detectable *Brucella* antibodies over time (Benavides et al., 2017).

For four feedgrounds with the most consistent serologic testing effort over time, we generated smoothed seroprevalence estimates using generalized additive models. We then calculated a calf:cow ratio for each site and year resulting in at least a decade of serological data per site. We modeled the number of calves per 100 adult female elk, *y*, as being normally distributed with a mean, *μ* and standard deviation, *σ*. The linear model for *μ* then consisted of an overall intercept, *α*_0_, with and without site‐varying intercept offsets, *α*_*j*_, and a fixed effect, *β*, for a particular serologic parameter, *ρ*. Thus, *μ* = *α*_0_ + *α*_*j*_ + *βρ*. We tested four different serological parameters, for a total of eight models. Accounting for the timing of transmission (abortion) events as well as the incubation period of brucellosis, we hypothesized that the change in seroprevalence from year *t−2* to year *t−1* as well as the change in seroprevalence from year *t−1* to year *t* should both strongly correspond to the rate of new infections immediately preceding periods when conception or abortion occurs which would be relevant for calf attendance in year *t*. We also hypothesized that herd‐level exposure would be sufficient to detect an effect and so used seroprevalence at year *t−2* and year *t−1* as predictors. We standardized all four of our serological predictors and used vague priors for all terms. The prior for the overall intercept, *α*_0_, was normally distributed with a mean of 20 and standard deviation of 20. The slope coefficient, *β*, was distributed normal with a mean of zero and standard deviation of 10. The standard deviations for the intercept terms were distributed half‐Cauchy with a location parameter of zero and scale parameter of 2.

In our pregnancy models, the prior for the overall intercept for the probability of pregnancy, *α*_0_, was drawn from a normal distribution with a mean of 2 and standard deviation of 1. Age‐ and year‐specific offsets, *α*_*j*_ and *α*_*k,*_ were given weakly informative normal priors with a mean of zero, and standard deviations which were drawn from a half‐Cauchy distribution with a location parameter of zero and scale parameter of 2. All other effects, including serostatus, pregnancy test method, time‐since‐infection, and FP, were given weakly‐informative normal priors with a mean of zero and standard deviation of 10. For models which featured varying intercepts and slopes for the age‐ and year‐varying effects of serostatus, a joint multivariate normal prior was used, $\left[\begin{array}{c} \alpha_{\mathrm{jk}}\\ \beta_{\mathrm{jk}} \end{array}\right]∼\text{MV}\;\;\;\text{Normal}\left(\begin{array}{c} \left[\begin{array}{c} 0\\ 0 \end{array}\right],\Sigma \end{array}\right)$. The mean vector consisted of zeros, while the covariance matrix, Σ, received hyperpriors and was further decomposed into a standard deviation matrix and correlation matrix, Ω, $\Sigma =\left(\begin{array}{c} \sigma_{\alpha_{\mathrm{jk}}}\\ 0 \end{array}\begin{array}{c} 0\\ \sigma_{\beta_{\mathrm{jk}}} \end{array}\right)\Omega \left(\begin{array}{c} \sigma_{\alpha_{\mathrm{jk}}}\\ 0 \end{array}\begin{array}{c} 0\\ \sigma_{\beta_{\mathrm{jk}}} \end{array}\right)$. Standard deviations were drawn from the same half‐Cauchy distribution previously used, $\left(\sigma_{\alpha_{\mathrm{jk}}},\;\sigma_{\beta_{\mathrm{jk}}}\right)∼\text{HalfCauchy}\left(0,\;2\right)$, while the correlation matrix received a relatively diffuse, unimodal Lewandowski, Kurowicka, and Joe (LKJ) prior, $\Omega ∼\text{LKJcorr}\left(2\right)$.

All models were fit in R version 3.3.3 (R Core Team, 2016) using the package rethinking (McElreath, 2016) and Stan (Stan Development Team, 2017). To quantify support for our models estimating the effect of serostatus on pregnancy probability, we used Widely Applicable Information Criterion (WAIC) (Vehtari, Gelman, & Gabry, 2017; Watanabe, 2010) and Akaike model weight. All models were run for 5,000 iterations after warm‐up with three chains. We assessed convergence by monitoring trace plots and using the Gelman‐Rubin statistic ($\hat{R}$). $\hat{R}$ values were all less than or equal to 1.1.

## 3 RESULTS

### 3.1 Effects of serostatus, test method, age and calendar day

Disease exposure status was the single best predictor of pregnancy in all of our models. Age was not by itself a strong predictor, but was important after accounting for disease status. Pregnancy test method also emerged as an important predictor in our models, with false‐positive ultrasound tests being the greatest source of error. Our models predicted a lower probability of pregnancy based on PSPB results than ultrasound (*ɣ *= −0.95, 89% HPDI −1.39, −0.52). This testing error did not vary by serostatus or age. Including age‐ and year‐varying intercepts further reduced WAIC of our models (Table 2).

**Table 2:** Model comparison for the effect of serostatus on pregnancy probability

Model	WAIC	pWAIC	dWAIC	Weight	*SE*	dSE
6	963.7	21.9	0.0	0.55	43.20	NA
7	964.1	25.4	0.4	0.45	43.25	1.71
5	977.3	19.5	13.5	0	43.12	7.69
4	989.0	8.2	25.2	0	44.02	11.28
3	1,003.3	6.0	39.5	0	44.00	13.67
2	1,018.3	16.0	54.6	0	43.05	16.59
1	1,042.4	2.9	78.7	0	43.42	19.09

*Note*

Model 6, including age‐ and year‐varying intercepts, age‐varying effect of serostatus, and effect of pregnancy test method had the lowest WAIC and received more than half of the Akaike model weight. Model 7, which differed from model 6 only in that it included year‐varying effects of serostatus, failed to improve model fit, and received the remainder of the Akaike model weight.

**Figure 2:**
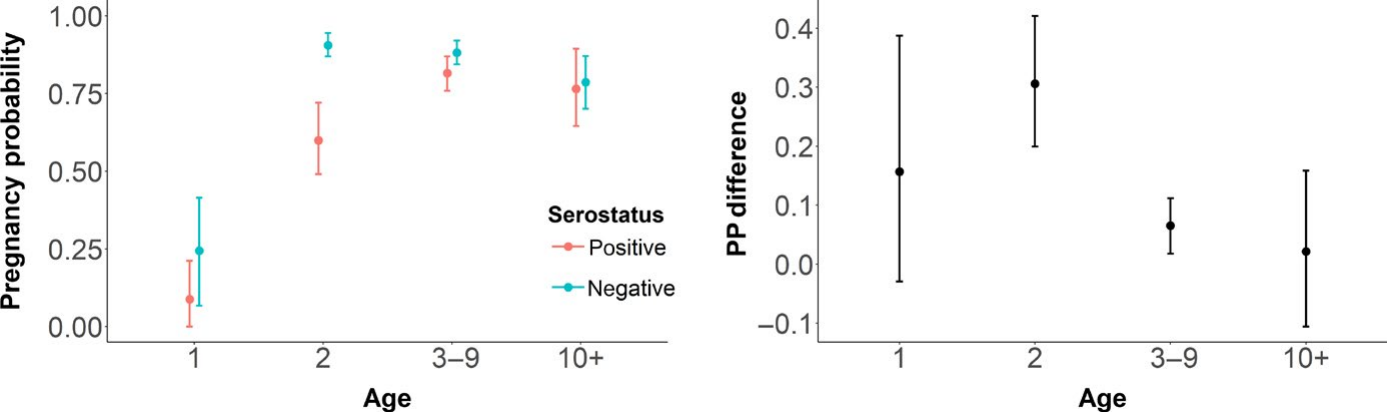
Left: Mean posterior predictive estimates of pregnancy probability by age group and serostatus from model 6, for the average year, with 89% highest posterior density interval (HPDI) estimates indicated. Right: The percentage point difference in mean estimates by age, for the average year, with 89% HPDI indicated. Mean estimates were 16% for yearlings, 31% for 2‐year‐olds, 7% for 3‐ to 9‐year‐olds, and 2% for animals 10 years of age and older

Being seropositive reduced the probability of pregnancy across all ages, with greater effects among younger elk. The largest percentage point difference was in the 2‐year‐old age class (Figure 2). Being seropositive reduced the probability of pregnancy by 31 percentage points for 2‐year‐olds (89% HPDI = 20–42 percentage point difference from seronegative mean) but only 7% for 3‐ to 9‐year‐olds (89% HPDI = 2%–11%). Relatively few pregnancy test results were available for yearlings, resulting in low precision of the estimates for this age class. The mean percentage point difference for yearlings was 16% (89% HPDI = −3% to 39%).

We found no evidence that the day of calendar year in which an individual pregnancy test was administered had any effect on the probability of pregnancy for seropositive elk (see Supporting information Appendix S1). The modeled effect for each standard deviation of the time covariate was 0.02, with a wide 89% credible interval (−0.30, 0.38).

### 3.2 Effect of year

**Figure 3:**
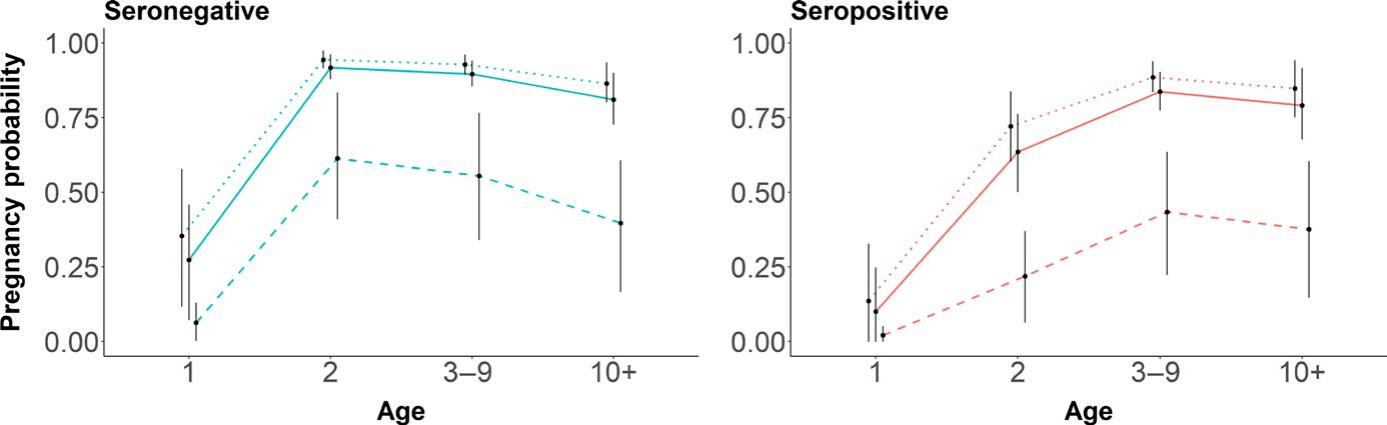
Mean posterior predictive estimates by age class of the probability of pregnancy for seronegative (left) and seropositive (right) elk in years of highest (2011, top line), average (2012, middle line), and lowest (2017, bottom line) overall pregnancy

Most years in our data exhibited similar levels of pregnancy, but there was considerable variation in pregnancy probability in a handful of years (Figure 3). In a post hoc analysis, we added year‐varying disease effects but found no evidence to suggest that the effect of disease changed over time or in years of relatively high or low overall pregnancy. Although the percentage point difference between mean estimates for the probability of pregnancy for seropositive and seronegative 2‐year‐old and 3‐ to 9‐year‐old elk increased in years of low overall pregnancy, these fell within the 89% HPDIs for age‐specific differences based on our top model (Figure 2).

### 3.3 Longitudinal data

For more than 500 seropositive records with FP assay values, there was weak evidence supporting the hypothesis that probability of pregnancy increases as *Brucella* antibodies decrease (see Supporting information Appendix S2). A decrease in one standard deviation of FP value increased pregnancy probability by <2% and the 89% credible interval for effect of FP value overlapped zero. Similarly, “time‐since‐infection” was a poor predictor of pregnancy. The mean estimates for the effect of time‐since‐infection were near zero, and these estimates remained unchanged whether or not age‐varying intercepts were included. In addition, for repeatedly sampled individuals, FP values declined at a rate of 15 mpu/year (89% HPDI = −19, −11). This appears to suggest that our metric for time‐since‐infection was adequate for testing recovery, despite not knowing with certainty when individuals were first infected. This finding also corroborates the idea that low FP results may be indicative of older exposures.

### 3.4 Calf:cow ratio

The change in seroprevalence from years *t*−*2* to *t*−*1* and the change in seroprevalence from years *t*−*1* to *t* had a negative association with the number of calves counted per 100 female elk in year *t*, and credible intervals did not overlap zero. Neither the seroprevalence at year *t*−*2* nor at *t*−*1* exhibited a statistically significant association with calves per 100 females. Allowing site‐varying intercepts reduced WAIC, but Akaike model weight was distributed across six of the eight models and none appeared to be a particularly good fit to the data (Supporting information Appendix S4). All four models including the “change in prevalence” predictors provided similar estimates. The top model predicts that for one standard deviation increase in prevalence from year *t*−*2* to *t*−*1* (approximately 6%) 1.6 fewer calves per 100 adult female elk would be counted in year *t* (89% HPDI = −0.31, −2.79).

## 4 DISCUSSION

Chronic disease impacts can be difficult to detect or go misdiagnosed because they typically require large, long‐term data sets. Our results show that, despite decades of research, there is a significant and previously undetected reduction in the probability of pregnancy in elk cows exposed to brucellosis. This effect appears to be additive to the abortions that the disease is notorious for, which effectively doubles the previously estimated reproductive cost of brucellosis to elk. We also found evidence that this hidden reproductive cost is long‐lived. It is uncertain, however, how this might influence population growth, in part because the mechanisms by which the disease might affect conception, implantation, and/or early pregnancy remain unidentified.

The estimated overall effect of reduced pregnancy on reproductive output, contingent on age‐specific prevalence and herd age structure, was 12 percentage points less than the seronegative mean (see Supporting information Appendix S3). We found substantial variation in effect size between age classes, which appeared to diminish in older age. This difference attributable to serostatus could result from failure to conceive during the previous rut or fetal loss, although we found compelling evidence that this reduction in pregnancy is not the result of abortions occurring during our sampling period. Because pregnancy is a prerequisite for abortion, the total reduction in reproductive output from this disease must be the sum of the two. By our estimation, this would suggest that seropositive elk produce approximately 24% fewer calves than seronegative elk (see Supporting information Appendix S3). Similar reductions in pregnancy and recruitment attributable to brucellosis have been found for bison in Yellowstone, which lend additional support to our findings (Fuller et al., 2007; Geremia et al., 2008). This indicates the potential for the disease to have as much of an effect on reducing pregnancy as severe winters or droughts.

A 24% reduction in reproductive output is substantial, but a few caveats must be considered. That value applies to seropositive females and, with periodic exceptions, it is the minority of elk within any given herd in our study area that test seropositive for brucellosis (Cotterill et al., 2018). Except in herds with particularly high seroprevalence, the expected decline in pregnancy probability across the herds we studied should generally be below 7%. In a typical GYE herd with 25% seroprevalence, in which the disease causes 12% of seropositive female elk to lose their reproductive opportunity by mid‐winter, and an additional 16% of seropositive elk go on to abort conditional upon being pregnant, then under average feedground conditions (76% of our seropositive elk were pregnant), the combined effect is a 6% reduction in healthy births as follows: (0.25 * [0.12 + (0.16 * 0.76)]  = 0.06). It must be noted, however, that local seroprevalence can at times exceed 60%, in which case, the expected overall reduction in calf production for the herd would be 14 percentage points lower. As we saw when we modeled the effect of year on pregnancy probability, in years when overall pregnancy rates are depressed for other reasons, the reproductive costs of brucellosis further compound low productivity. While we did detect a decrease in calf:cow ratio attributable to increases in seroprevalence, the observed effect was weaker than expected. This could be because the calf data included sampling error arising from the use of ground counts on highly aggregated herds and also because changes in smoothed seroprevalence estimates are imprecise. A thorough accounting of other variables that influence recruitment to calfhood was simply beyond the scope of this study.

The mechanism behind the observed reductions in pregnancy remains unidentified. Robust longitudinal data would add to our understanding of the cause, but are costly and difficult to obtain. A captive study would be ideal, but is ruled out in the United States by the Select Agent Status imposed on live *Brucella* cultures (National Academies of Sciences, Engineering, and Medicine, 2017). We were unable to resolve this issue, but our analysis of the available repeat‐capture data suggests that female elk incur longer‐term reproductive consequences of this disease than previously thought. Both “time‐since‐infection” and FP assay were poor predictors of pregnancy probability for seropositive elk, which does not support the conventional belief that elk recover fully from this disease after the initial abortion risk has passed. Chronic inflammation of the endometrium (endometritis) has been described across the *Brucella* genus in other hosts, which could interfere with implantation (Enright, 1990; Meador et al., 1988; Rhyan et al., 2009; Verma et al., 2000). Although this seems a plausible biological cause, it does not fit well with the large effect in 2‐year‐olds, most of which are expected to be pregnant for the first time.

These findings complicate studies of GYE elk which have shown reduced recruitment over time but failed to take brucellosis into account. In some instances, data may exist to show the disease was absent or at very low levels for the herds in the question, while in others, there may simply be no relevant disease data. Reduced fecundity because of disease may also add complexity to disease dynamics. When time‐series prevalence data are used to evaluate disease‐management strategies, then differential vital rates must be taken into account along with other potentially relevant factors like seasonality of transmission (Altizer et al., 2006), climate (Pascual et al., 2008), and cohort entry effects (He & Earn, 2016). A chronic disease like brucellosis, which affects fecundity, might provide an excellent model system in which to study the fitness consequences of disease and the evolution of disease resistance or tolerance. This line of inquiry is particularly relevant now with the spread of chronic wasting disease (CWD), a spongiform encephalopathy that affects cervids, causing global concern (Galloway et al., 2017; Mysterud & Rolandsen, 2018). It has been suggested that selection for prion disease resistance may play an important role in the long‐term dynamics of infected populations as CWD continues to spread (Monello et al., 2017; Robinson et al., 2012; Williams et al., 2014). Similarly, the magnitude of the effect of brucellosis on elk fitness should drive selection for increased resistance or tolerance. Whereas CWD is eventually fatal for elk (Williams et al., 2008), the fitness consequences of brucellosis take effect much sooner (although they may also be prolonged). If coinfection becomes common in GYE elk then an intriguing question will arise: could selection for resistance to one disease disrupt selection for resistance to the other?

Lastly, the operation of supplementary feedgrounds for wildlife is a subject of perennial debate and, for the GYE elk in particular, the brucellosis problem places feedground managers on the horns of a dilemma. First, elk‐cattle transmission risk is reduced by the spatial separation achieved by feedgrounds (Brennan et al., 2017), but feedgrounds increase elk–elk transmission while maintaining high local prevalence (Cross et al., 2007) and serving as a source to elk that are remote from feedgrounds (Kamath et al., 2016). Second, while feedgrounds increase the reproductive cost of brucellosis, they may also offset them by nutritional supplementation of the elk cows that proceed to healthy parturition. The proportion of seronegative feedground elk that are pregnant rival some of the higher reported pregnancy rates for Rocky Mountain populations (Raithel et al., 2007). Consequently, at the scale of the GYE, the overall effect of brucellosis on population growth appears benign under normal conditions for nutritionally supplemented elk. Closing feedgrounds while brucellosis prevalence is high might result in poor recruitment in the near term, particularly if nutritional supplementation has been offsetting disease costs. Because brucellosis in elk has historically been considered a “feedground problem,” and feedgrounds facilitate handling and sampling opportunities, the bulk of the ecological research surrounding it has taken place within these fed herds. The full effects of brucellosis remain to be tested in unsupplemented elk herds where food resources, herd demographics, local force‐of‐infection, and other factors differ. With brucellosis prevalence increasing among many elk herds in the GYE and beyond (Cross et al., 2010; National Academies of Sciences, Engineering, and Medicine, 2017), brucellosis and other disease impacts should be an important consideration in the future demographic studies. Failure to account in full for disease‐induced reproductive costs could cause over‐estimates of the effects of other demographic stressors, all of which require different management and policy strategies.

## AUTHORS’ CONTRIBUTIONS

PCC, ADM, JDR, and BMS conceived the idea for this article. Data acquisition was performed by JDR and BMS. Data analysis was primarily conducted by GGC with help from PCC. The article was primarily written by GGC and JTdT. All authors contributed critically to the drafts and gave final approval for publication.

## DATA ACCESSIBILITY

Data and supporting code available through the Utah State University Digital Commons: https://doi.org/10.15142/t39m0b.
