# Supplementary material for: Hidden cost of disease in a free‐ranging ungulate: brucellosis reduces mid‐winter pregnancy in elk
Source: Ecol Evol. 2018 Oct 28;8(22):10733–42. doi: 10.1002/ece3.4521 (PMC6262735; doi:10.1002/ece3.4521)
Supplement: Supplementary file 1 [file ECE3-8-10733-s001.docx]

**Appendix** **S1**

**Does the probability of pregnancy among seropositive female elk decrease over the sampling period?**

From previous work we expected that February was the earliest possible time that abortions could occur (Paul C. Cross et al., 2015), but that they should not happen with any great frequency until March, April, and May.

Pregnancy test results were performed (or blood sample was collected) between January and April at feedgrounds for any given year. 90% were collected prior to March 1. If the difference in pregnancy between seropositive and seronegative elk is in part (or entirely) due to disease-induced abortions, then, using seropositive pregnancy test data, we would expect a decreased probability of pregnancy later in the sampling period. Plotting the raw pregnancy data for seropositive elk provides a sense of data distribution (Fig. A1).
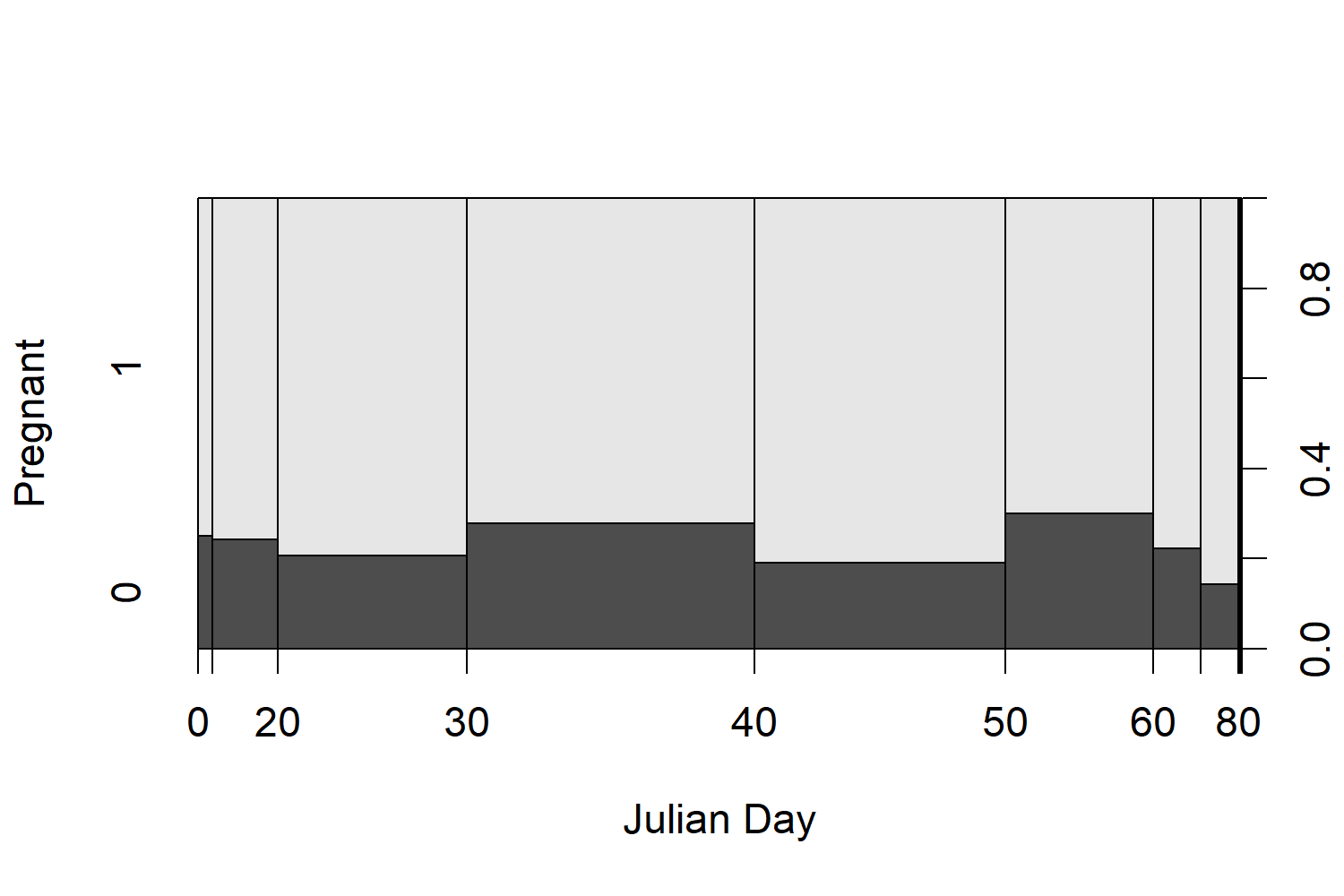


Figure A1. Proportion of the sample that was pregnant by 10-day period of the calendar year (n=587). The width of the bars reflects sample size per 10-day window. Almost all of the data was collected in the 40-day period between January 20th and March 1st. Approximately 80% of the sample were pregnant (lighter shading where ‘pregnant = 1’ corresponds to pregnant).

We tested for a significant change in pregnancy over the sampling period with 3 models. First, a generalized linear model where, $\alpha_{0}$ is the intercept for the probability that a seropositive elk is pregnant and $\beta_{0}$ is the slope coefficient for the time covariate, $\zeta_{i}$.:

$$logit(p_{i})=\alpha_{0}+\beta_{0}\zeta_{i}$$

The second model incorporated an age-varying intercept, while the third incorporated an age-varying intercept, $\alpha_{j}$, and age-varying slope, $\beta_{j}$:

$$logit(p_{ij})=\alpha_{0}+\alpha_{j}+(\beta_{0}+\beta_{j})\zeta_{i}$$

We standardized Julian day by subtracting the mean and dividing by the standard deviation. Model 3 received the lowest WAIC and the majority of the model weight (table A1).

Table A1. Model results for the effect of Julian day on the probability of a seropositive elk being pregnant. Model 3, which included an age-varying intercept and slope, was the top model.

| model | intercept | slope | WAIC | weight |
| --- | --- | --- | --- | --- |
| 3 | age-varying | age-varying | 626.7 | 0.65 |
| 2 | age-varying | fixed | 627.9 | 0.35 |
| 1 | fixed | fixed | 648.8 | 0.00 |
|  |  |  |  |  |
|  |  |  |  |  |

The overall beta estimate from this model is 0.02 (89% HPDI = -0.30, 0.38). The point prediction of the model corresponds to a 2 percentage point increase in the probability of pregnancy for seropositive elk between early January and mid-April, however the credible intervals are very wide and overlap zero. Thus we have no evidence to suggest the probability of pregnancy changes over the sampling period.

These model results suggest that the difference in pregnancy probability between seropositive and seronegative elk is either due to reduced rates of conception among seropositive elk, or intrauterine mortality prior to January. In either case, this appears to be a separate phenomenon from the disease-induced abortions which occur annually among ~16% of seropositive and pregnant elk.

**Appendix S2**

**Do lower fluorescent polarisation assay (FP) test results correspond to higher pregnancy probability in seropositive elk?**

In our main models there was a larger apparent effect of serostatus on pregnancy for young animals. We are interested in why that might be, and what our data can suggest about a possible mechanism.

Antibodies for brucellosis in elk are retained for many years but can eventually be lost (Benavides et al., 2017). If they are not lost, then seroprevalence as a function of age should follow Muench's catalytic model (Muench, 1959). As a result, age is generally correlated with disease exposure and time-since-infection. Elk could recover from the reproductive consequences of the disease, but remain seropositive, which is possibly why the effect of serostatus on pregnancy is attenuated at older age. In other words, the recency with which the infection was acquired may be more relevant than age. FP scores give us a continuous measure of serology to work with, so a reasonable question to ask is whether seropositive elk are more likely to be pregnant when FP scores are lower.

Our sample size for this analysis was 510 records. The GLM, $logit(p_{i})=\alpha_{0}+\beta_{0}\eta_{i}$, where $\eta_{i}$ corresponds to the standardized FP value of individual, $i$, gives us a beta estimate of -0.07 and CIs overlap zero (89% HPDI = -0.25, 0.09). The point estimate translates to a 1% increase in the probability of pregnancy for each decrease in one standard deviation of FP assay score, but with wide credible intervals that overlap zero these results do not appear significant. The 89% HPDI estimates correspond to a span from a 5% increase to 2% decrease in pregnancy probability per one standard deviation of FP score decrease and the standardized values of FP score in our data ranged from -2.8 to 2.1.

**Does the probability of pregnancy increase as time-since-infection increases?**

Based on the repeat capture of marked individuals with repeat serology and pregnancy test results we were able to generate 242 records where we knew the year in which the individual elk first tested seropositive. The sampling leaves a lot to be desired, and sample sizes are as follows:

| Time since first seropositive test | 0 | 1 | 2 | 3 | 4 | 5 | 6 | 7 | 8 | 9 | 10 | 11 | 12 | 13 |
| --- | --- | --- | --- | --- | --- | --- | --- | --- | --- | --- | --- | --- | --- | --- |
| Sample size | 134 | 46 | 35 | 6 | 7 | 7 | 3 | 0 | 1 | 1 | 0 | 0 | 1 | 1 |

These observations represent 134 unique individuals.

Thorne et al. (1978) reported an average incubation period of 39 days following artifical infection to become seropositive, and due to the timing of abortions (transmission) we strongly suspect that in almost all cases individuals can only first be detected as seropositive in the winter following the winter/spring in which they contracted the infection. As such, 0 'time since first seropositive test' should equate to 1 year since becoming infected. And indeed the proportion of those records that were pregnant is 77%, which is very similar to what we expect from the data for all seropositives, ignoring age. By contrast, if these reflected individuals which had become infected in the previous month or two, the proportion that are pregnant should be closer to 90%.

With this new dataset, we can also test whether FP scores actually decrease with time the way we anticipated. We end up with 212 observations to test this notion. This is a simple linear model $FP\sim Normal(\mu,\sigma),$ and $\mu=\alpha+\beta\delta$, where $\delta$ is the 'time since first seropositive test' covariate. Our intercept ends up being 218, with a beta estimate of -15.09 (89% HPDI = -18.95, -11.02). This looks significant and supports the hypothesis that lower FP scores may be indicative of longer time since infection. Still, caution is warranted lest we read too much into FP values.

A generalized linear model gives us a beta estimate for the effect of time-since-infection of -0.01 with wide credible intervals (89% HPDI = -0.15, 0.13). This intercept translates into a 77% probability of pregnancy and an estimated 2% increase in the probability of pregnancy for each year afterwards, but the effect is not significant. This does not lend support for the idea of recovery. Although these data are imperfect, we do have >30 samples for the 1st, 2nd, and 3rd years following first seropositive test.

The conventional belief among brucellosis/elk/feedground researchers since Thorne et al.’s captive study in the 1970s was that some elk experience reproductive failures in the year or two following infection, and afterwards emerge recovered and relatively unscathed. We expected that the same would hold true for what appears to be 'failure to conceive', but so far we have not found evidence to support that belief.

**Appendix S3**

**Estimating a ‘total apparent effect’ of serostatus.**

Method 1: “Accounting for herd age structure and age-specific prevalence”

One way to do this is to calculate an average based on the age-specific prevalence curve (Fig. C1), some population structure (Fig. C2), and the point estimates from the age-specific percentage point differences in pregnancy probability by serostatus (from our top model). Age-specific prevalence follows that reported in Benavides et al. (2017). Here we used all known-age data from the Wyoming feedgrounds and fit a smoother.


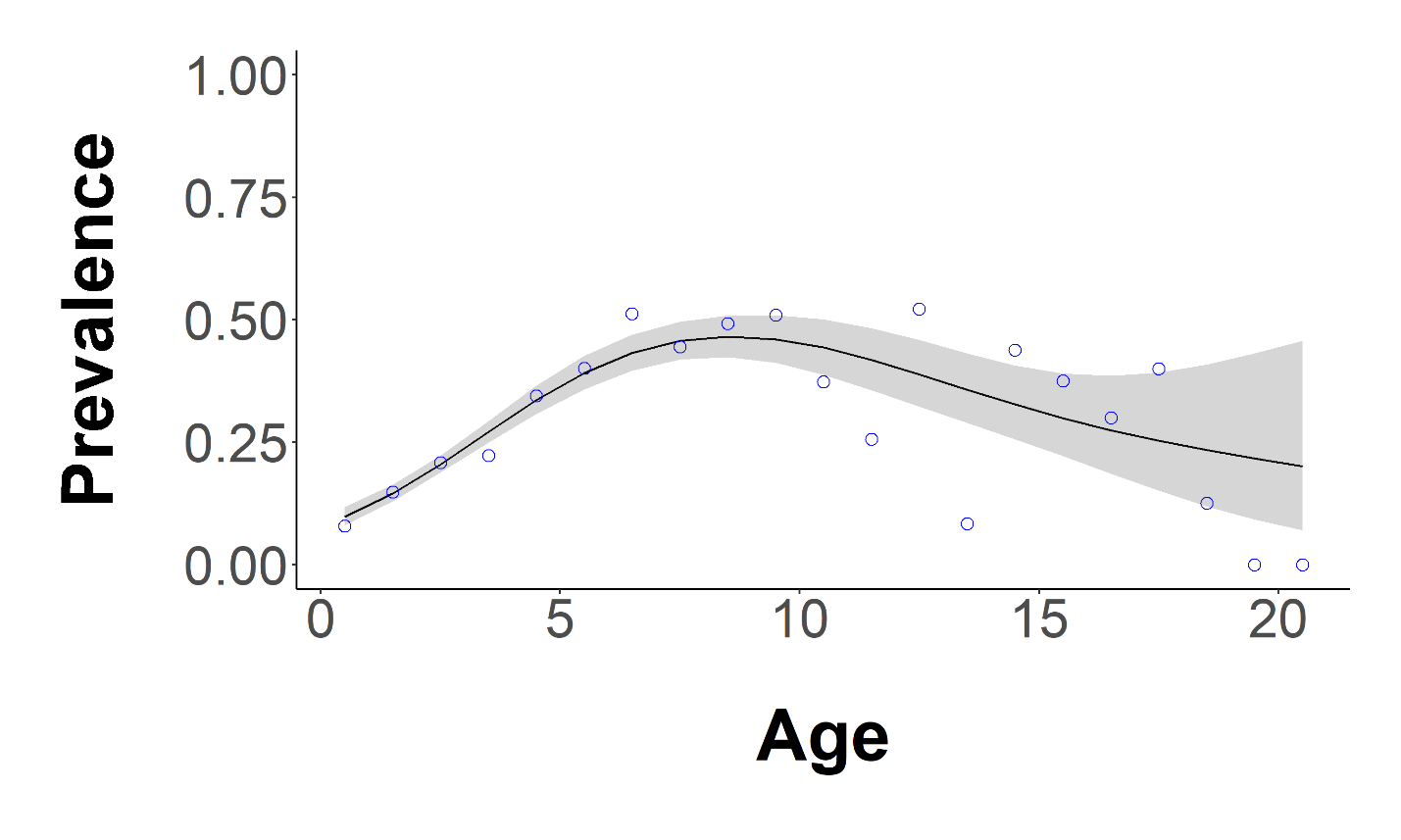


Figure C1. Age-specific seroprevalence for brucellosis of Wyoming feedground elk. Open blue circles represent raw data, while the black line represents model estimates from a generalized additive model, and the shaded area represents 95% confidence intervals.


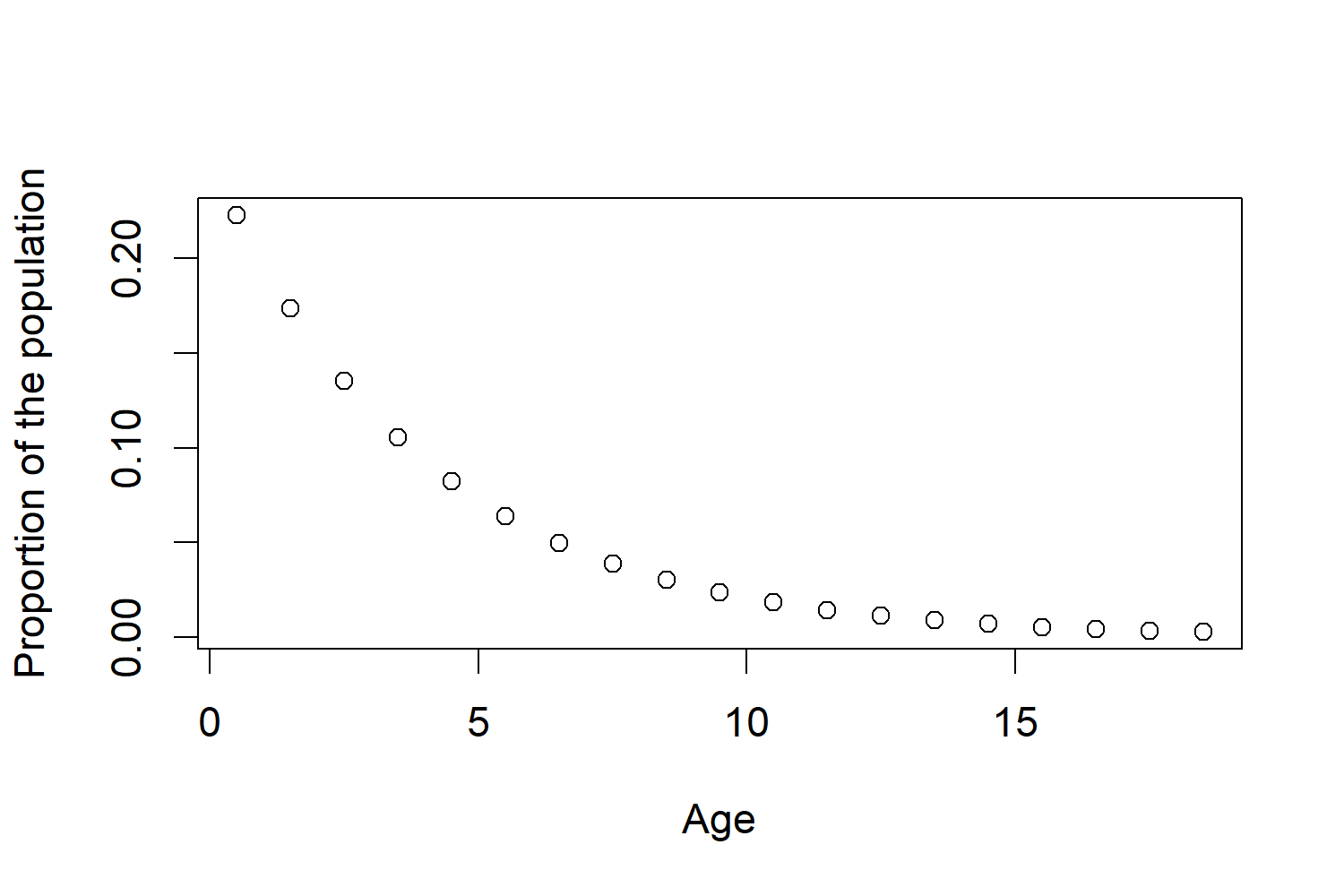


Figure C2. A simulated population structure for elk spanning from 6 months old to 18 ½ years old. Dots represent the proportion of the total population belonging to each age.

An approximate population structure was simulated using the density of the exponential function with a rate of 0.25, which fit the distribution of our known-age data well. The point estimates for the difference in pregnancy probability (the percentage point difference from the seronegative mean) were 0.16 (yearling), 0.31 (2-year-olds), 0.7 (3- to 9-year-olds), and 0.2 (10+).

If, for each age from 6 months to 18 ½ years, we multiply the age-specific prevalence by the proportion of the population by the expected difference in pregnancy probability and take the average, we arrive at 0.12, or 12 percentage points fewer than than the seronegative expectations.

Method 2: accepting an average effect of serostatus on pregnancy that includes some age-bias

Ignoring the age-varying effects of serostatus but keeping age-varying intercepts (as in model 3), we can calculate the average expected effect across all ages, again using point estimates. The percentage point differences from model 3 end up being 13.4% (yearling), 12.9% (2-year-olds), 11% (3- to 9-year-olds), and 16% (10+). The weighted average for all ages up to 18 ½ years old is then 13.7%.

**Appendix S4**

Table D1. Model rankings for 8 models to predict the number of calves counted per 100 adult female elk in year *t* by serological parameters. Increases in seroprevalence at a site in the last 1 or 2 years were associated with fewer expected calves, shown here with 89% HPDI.

| rank | intercepts | serological parameter | WAIC | weight | estimated effect |
| --- | --- | --- | --- | --- | --- |
| 1 | site-varying | $\Delta$ *t-2* to *t-1* | 450.7 | 0.41 | -1.60 (-2.79, -0.31) |
| 2 | site-varying | $\Delta$ *t-1* to *t* | 451.9 | 0.23 | -1.39 (-2.64, -0.10) |
| 3 | site-varying | prevalence *t-1* | 452.9 | 0.14 | -0.90 (-2.16, 0.38) |
| 4 | site-varying | prevalence *t-2* | 453.7 | 0.09 | -0.30 (-1.51, 1.03) |
| 5 | fixed | $\Delta$ *t-2* to *t-1* | 454.1 | 0.08 | -1.98 (-3.24, -0.74) |
| 6 | fixed | $\Delta$ *t-1* to *t* | 455.3 | 0.04 | -1.83 (-3.04, -0.59) |
| 7 | fixed | prevalence *t-1* | 459.5 | 0.00 | -0.89 (-2.17, 0.39) |
| 8 | fixed | prevalence *t-2* | 461.1 | 0.00 | -0.07 (-1.38, 1.22) |


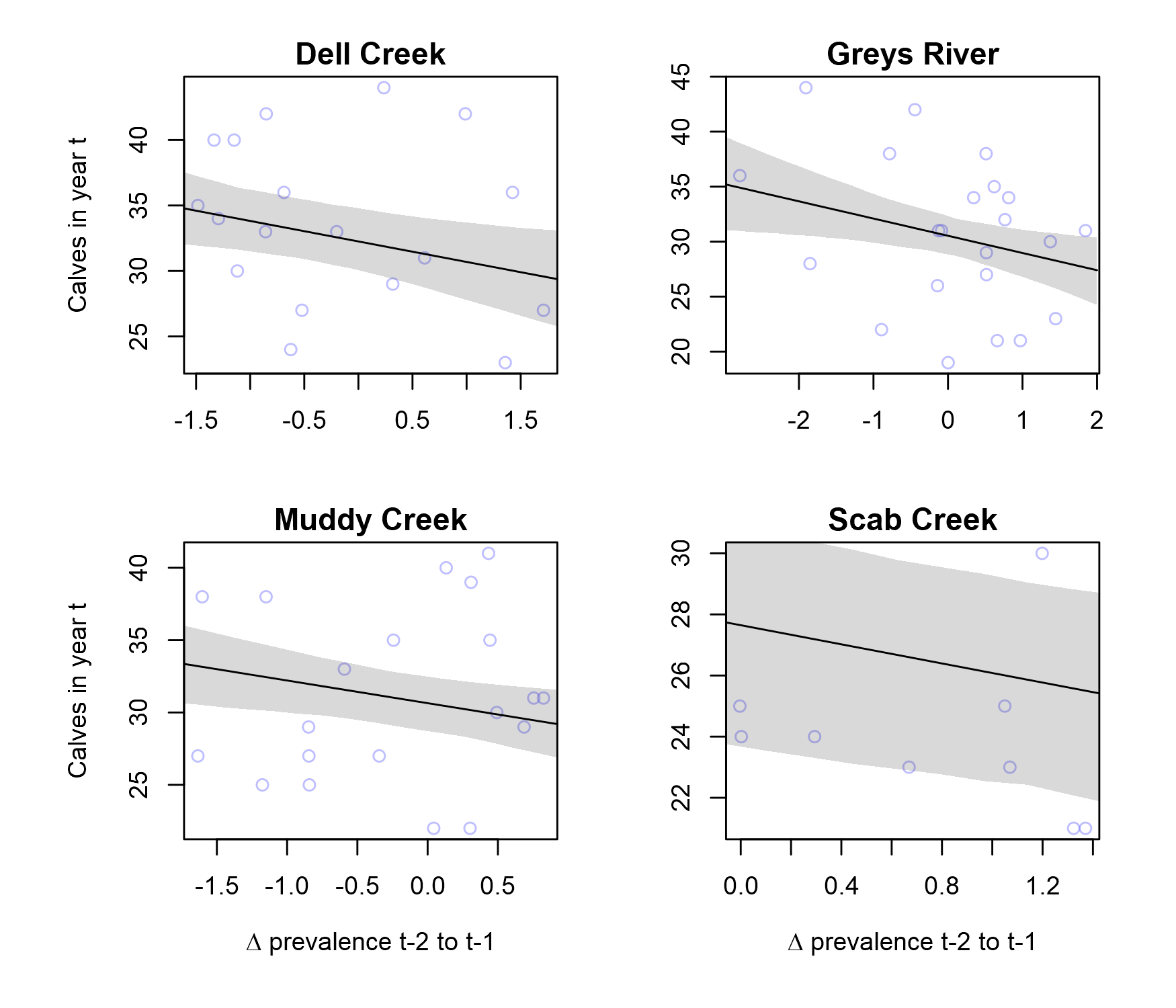


Figure D1. Results from the top model for the effect of serological parameters on the number of calves present during peak winter per 100 adult female elk. The solid black lines represent the mean estimate for the effect of a change in seroprevalence from year *t-2* to *t-1* (standardized), while the shaded portion represents the 89% highest posterior density interval. Allowing the intercept to vary by site modestly improved model fit. The model predicts 1.6 fewer calves per 100 adult female elk in year *t* for an increase in one standard deviation of seroprevalence (5.8%) from year *t-2* to *t-1*.


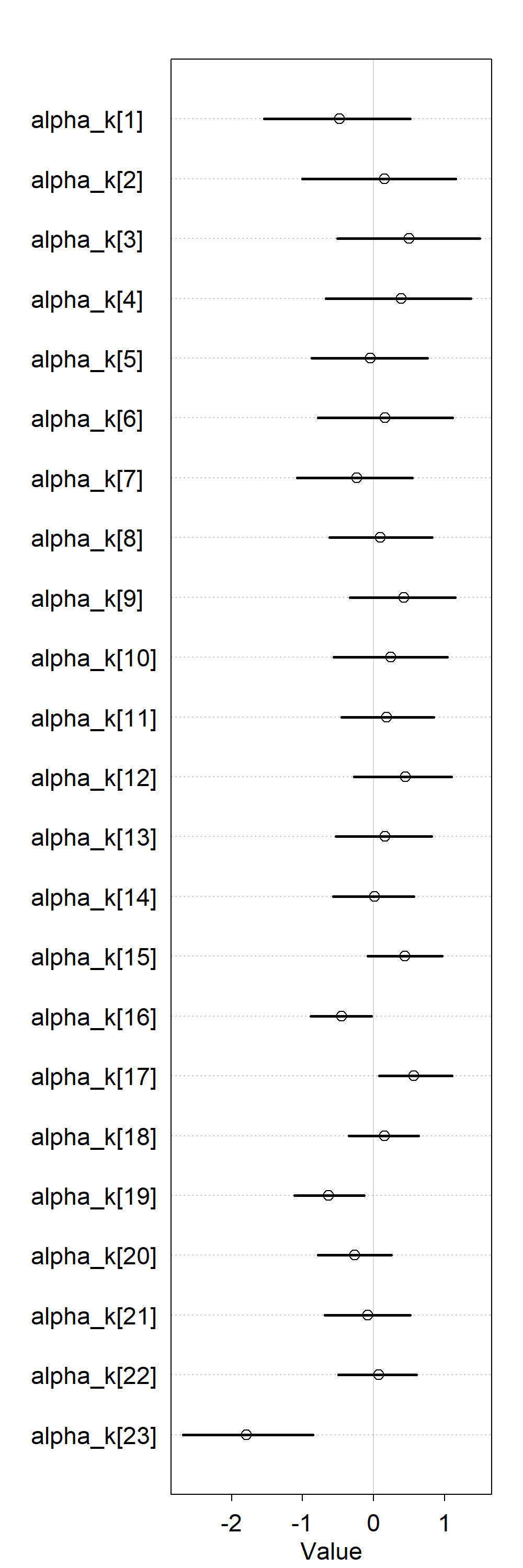


Figure D2. Estimates for the year-varying intercepts from the top model for pregnancy. In only 4 of 23 instances does it appear that a year has been significantly different from the mean in terms of overall levels of elk pregnancy at the feedgrounds. Alpha_k[23], which corresponds to 2017, is the outlier.
